# A cheesy exudate: a case report on management of pericardial disease through multimodal cardiac imaging

**DOI:** 10.1093/ehjcr/ytag261

**Published:** 2026-04-16

**Authors:** Aadavan Elangovan, Thomas Lewandowski, Dhaval Naik, John Spratt, Hussain Khalid

**Affiliations:** University of Florida, College of Liberal Arts and Sciences, 330 Newell Drive, Gainesville, FL 32603, USA; Division of Cardiovascular Medicine, Department of Medicine, University of Florida Health, 1600 SW Archer Rd, Gainesville, FL 32608, USA; Division of Cardiovascular Medicine, Department of Medicine, University of Florida Health, 1600 SW Archer Rd, Gainesville, FL 32608, USA; Division of Cardiovascular Medicine, Department of Medicine, University of Florida Health, 1600 SW Archer Rd, Gainesville, FL 32608, USA; Division of Cardiovascular Medicine, Department of Medicine, University of Florida Health, 1600 SW Archer Rd, Gainesville, FL 32608, USA

**Keywords:** Cholesterol pericardial effusion, Imaging, Case report, Pericardial constriction

## Abstract

**Background:**

Cholesterol pericardial effusion is a rare diagnosis and typically not associated with the development of pericardial constriction. However, covariate factors, such as Rheumatoid arthritis, myxoedema, and comorbid disease requiring anticoagulation, can predispose to developing dense adhesions that ultimately can cause pericardial constriction and constrictive physiology. This can develop without preceding symptoms but ultimately may declare itself with a variety of clinical presentations including syncope.

**Case summary:**

We present a patient with a pericardial mass incidentally identified during hospital evaluation after presentation with syncope. Initial suspicion based on computed tomographic angiography (CTA) appearance was of a pericardial cyst. Additional multimodal imaging consisting of transthoracic echocardiogram (TTE) and magnetic resonance imaging (MRI) supported evidence of a right ventricular–compressive effusion with constrictive physiology, findings more suggestive of a loculated pericardial effusion than a pericardial cyst without evidence of pericardial inflammation. Although transoesophageal echocardiography (TEE) guided pericardiocentesis was technically successful, it failed to produce clinical or haemodynamic improvement of the pericardial constriction. Partial local pericardiectomy and cruciate incision of the anterior aspect of the rind was essential in relieving the constrictive process. Pathologic analysis of resected pericardium demonstrated cholesterol granuloma. We discussed the role of multimodal imaging in disease classification, as well as coexisting conditions such as rheumatoid arthritis and anticoagulation for pulmonary embolism that contributed to this case’s unique presentation and timeline.

**Discussion:**

This case underscores the critical role of a multimodality imaging strategy in pericardial disease—particularly in managing rare cholesterol loculated effusions—and highlights how coexisting conditions can shape disease progression and treatment timing.

Learning points

- Cholesterol pericardial effusion is rare and frequently idiopathic. Associated conditions include Rheumatoid arthritis, myxoedema, mycobacterium tuberculosis infection, hyperlipidaemia, and trauma.
- Multimodality imaging allows anatomical delineation, assessment of haemodynamic effects, and tissue characterization to aid diagnosis and treatment of pericardial disease.
- For loculated effusions with mass effective and constrictive features without a predominantly anterior location, surgical management may be required.

## Introduction

Cholesterol pericardial effusion is rare, and presentation as a loculated pericardial effusion with constrictive physiology is uncommon.^1,2^ Multimodality cardiac imaging is essential in the management of effusive-constrictive pericardial disease.^3^ Although frequently idiopathic, cholesterol pericardial effusion has been associated with comorbid conditions such as rheumatoid arthritis, myxoedema, mycobacterium tuberculosis infection, hypercholesterolaemia, and trauma or haemorrhage.^1^ We present a case that highlights the role of multimodality cardiac imaging in guiding the diagnosis and treatment of a patient presenting with symptoms due to a rare aetiology of loculated pericardial effusion.

## Summary figure

**Figure:**
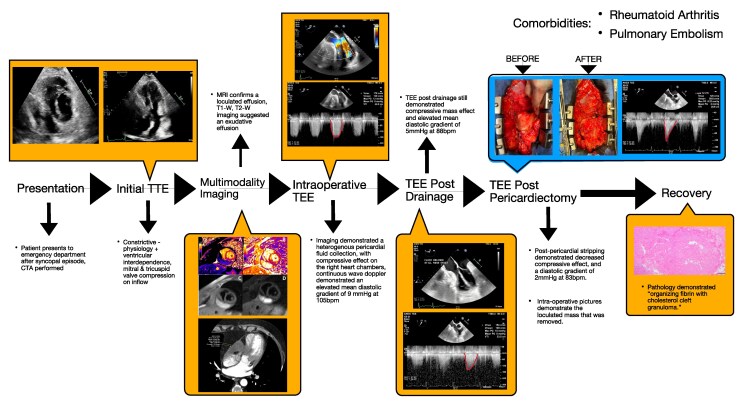


## Case presentation

We present a 36-year-old male with essential hypertension, Rheumatoid arthritis (RA), and recently diagnosed pulmonary embolism (PE). A month prior, he had presented to an outside hospital after a syncopal episode. He was diagnosed with acute PE and started anticoagulation with Apixaban. In addition to the PE, CTA of his chest demonstrated a small pericardial fluid collection thought to be a pericardial cyst.

**Figure 1:**
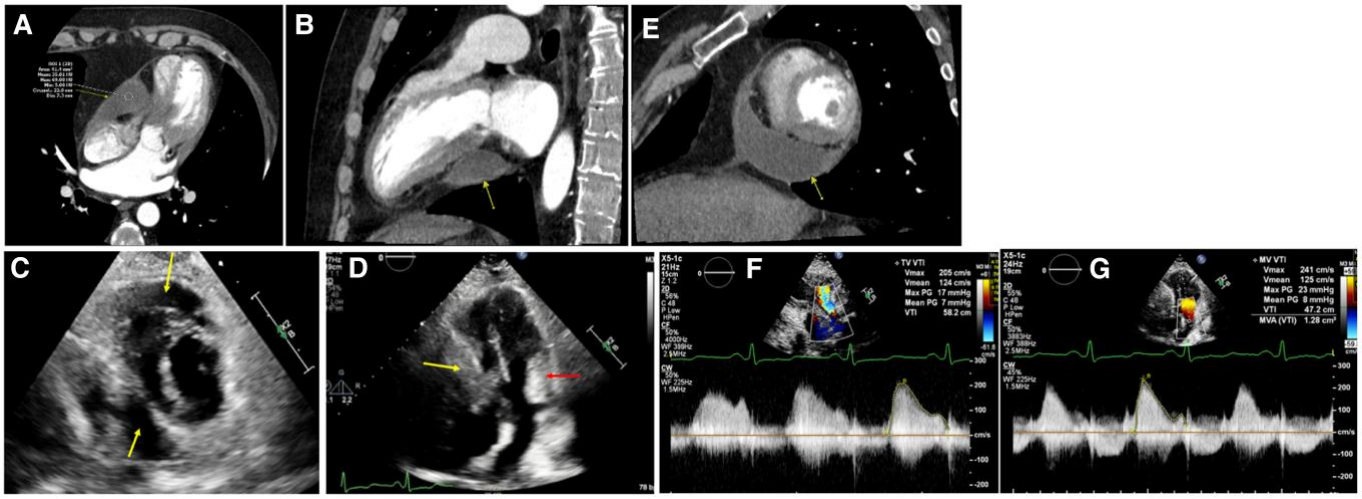
Cardiac CTA demonstrated a loculated pericardial effusion (arrows) exerting mass effect on the right atrium and ventricle, and to a lesser degree the left-sided chambers, with mean attenuation of 36 Hounsfield units suggesting a non-simple fluid collection (panels *A*, *B*, *E*). TTE confirmed chamber compression by the echolucent pericardial mass (arrows) with respiratory septal shift indicating ventricular interdependence, and additional compression of the mitral inflow near the lateral annulus (arrow, Panels *C*, *D*). Continuous-wave Doppler revealed elevated velocities and mean diastolic gradients across the tricuspid (mean diastolic gradient 7 mmHg at 75 bpm) and mitral valves (mean diastolic gradient 8 mmHg at 75 bpm), consistent with extrinsic compression from the pericardial mass (Panels *F*, *G*).

**Figure 2:**
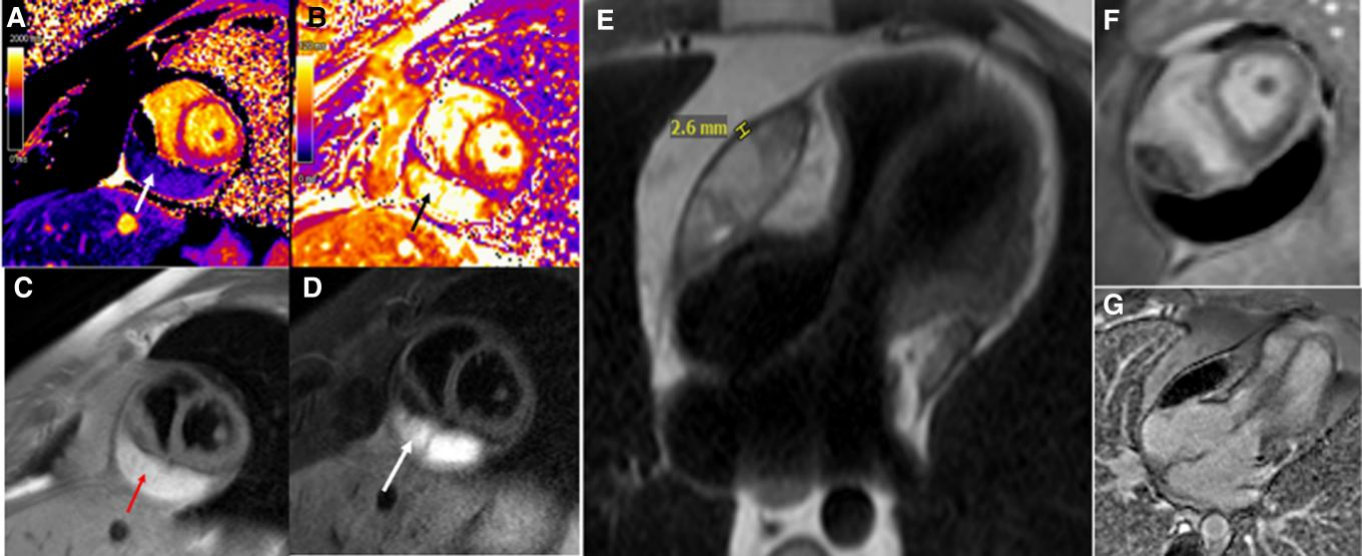
Cardiac MRI demonstrated a pericardial fluid collection with T1 mapping showing shorter relaxation time than myocardium (panel *A*) and corresponding hyperintensity on T1-weighted fat-suppressed imaging (panel *C*). T2 mapping revealed a longer relaxation time than myocardium (Panel *B*) with hyperintensity on T2-weighted STIR imaging (Panel *D*), without evidence of pericardial oedema. A loculated pericardial effusion with maximum pericardial thickness of 2.6 mm was visualized (Panel *E*). Phase-sensitive inversion recovery late gadolinium enhancement sequences showed no myocardial or pericardial enhancement (Panels F, G).

On the day of presentation the patient attended an outdoor event, experienced light-headedness and sustained another syncopal episode. The patient had a repeat CTA chest demonstrating a reduction in PE burden, but a persistent pericardial fluid collection with evidence of compression of the right ventricle (*Figure 1*). TTE demonstrated local constrictive/compressive features associated with the pericardial fluid collection, again, initially thought to be a pericardial cyst due to its proximity to the right ventricle (RV) (*Figure 1C, D*). An echolucent collection was visualized lateral to the RV free wall adjacent to the tricuspid valve annulus and wrapping interiorly/posteriorly around the RV and extending to abut the inferior left ventricular (LV) wall. Constrictive physiology was evident; biventricular compression at the level of the AV groove resulting in elevated diastolic gradients across the tricuspid and mitral valves and respiratory variation in the ventricular septal motion suggestive of ventricular interdependence were observed (*Figure 1F, G*). Cardiac MRI (CMR) demonstrated a hyperintense pericardial fluid collection on both T1- and T2-weighted imaging (*Figure 2A-D*) sequences suggestive of an exudative effusion.^4^ No late gadolinium enhancement (LGE) of the pericardium was seen, suggesting the absence of an active inflammatory process (*Figure 2F, G*). CMR perfusion sequences suggested the pericardial mass was non-vascularized.

Evacuation of the collection was sought due haemodynamic significance demonstrated on multimodality imaging and the persistence of orthostatic intolerance which the patient had not previously experienced. Anti-inflammatory therapy was deferred given the lack of pericardial inflammation by MRI. Pericardiocentesis was thought to be unfeasible and a suboptimal approach because of the anatomical location and contents of the collection, and significant localized constrictive physiology. Surgical consultation was requested for consideration of operative drainage.

**Figure 3:**
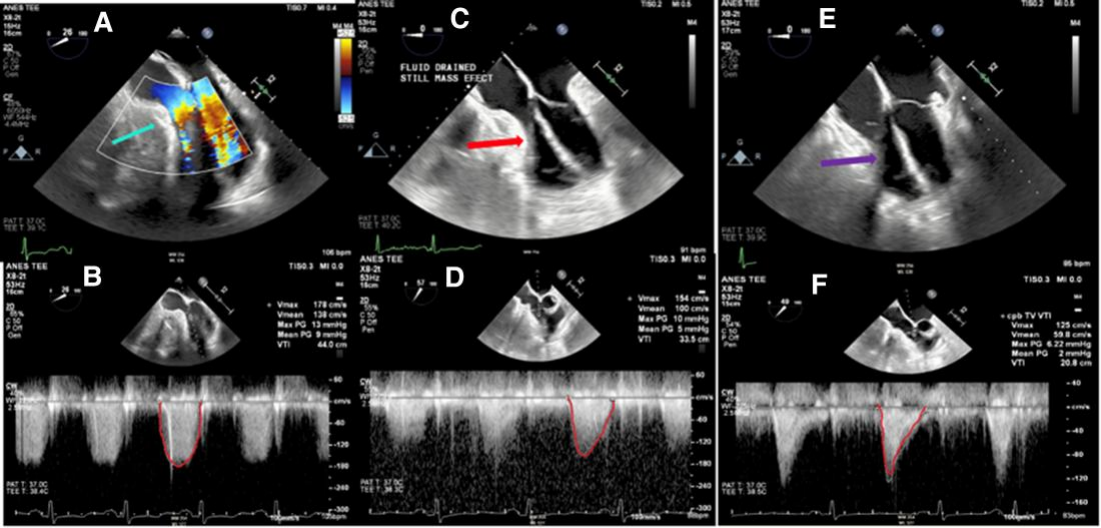
Intraoperative transoesophageal echocardiography (TEE) before and after pericardial interventions. Prior to drainage, a heterogeneous pericardial fluid collection (arrow, Panel *A*) was seen adjacent to the right-sided chambers with compressive effect, accompanied by an elevated tricuspid mean diastolic gradient of 9 mmHg at 105 bpm (Panel *B*). After drainage, the compressive mass effect persisted (arrow, Panel *C*) with only partial improvement in haemodynamic, as the tricuspid mean gradient remained elevated at 5 mmHg at 88 bpm (Panel *D*). Following pericardiectomy, there was clear decompression of the right-sided chambers (arrow, Panel *E*) with normalization of haemodynamic, and the mean diastolic gradient reduced to 2 mmHg at 83 bpm (Panel *F*).

**Figure 4:**
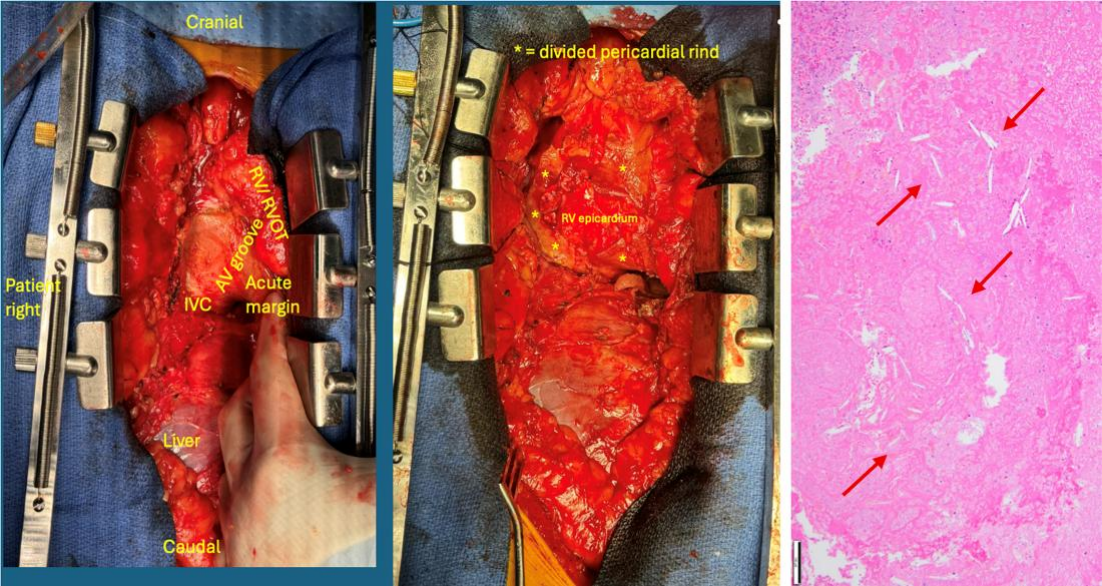
Intraoperative views during sternotomy demonstrating dense inflammatory adhesions and a constrictive pericardial rind encasing the heart (left). Following cruciate incision, the divided pericardial rind is shown with exposure of the right ventricular epicardium (centre). Histopathology (right) revealed organizing fibrin with cholesterol cleft granuloma, consistent with (arrows), consistent with cholesterol pericardial disease.

In the operating room prior to drainage, TEE re-demonstrated constrictive physiology (*Figure 3A*). A pericardial window was performed. Although typically performed via an intercostal approach, a subxiphoid approach was used given the possible need to convert to pericardiectomy. This yielded dark serous fluid, with ‘cottage cheese-like caseous’ exudate. Intraoperative TEE demonstrated persistent RV constriction despite drainage (*Figure 3C, D*). The subxiphoid incision was converted to a full sternotomy in anticipation of pericardiectomy. There appeared to be a dense, constricting rind covering the anterior surface of the right atrium and right ventricle, extending inferiorly along the anterior aspect of the inferior vena cava and posteriorly to the inferior wall of the LV (*Figure 4*). There was dense chronic inflammatory adhesion between the pericardium and the RV epicardium that prevented complete local pericardiectomy but ultimately the RV compression was relieved via cruciate incision of the anterior aspect of the rind. Following this, the TV gradient was significantly decreased 2 mmHg at heart rate 70 beats per minute from 9 mmHg at heart rate 72 beats per minute originally (*Figure 3E, F*). The cytology of the pericardial fluid was negative for malignancy and pathologic analysis of the pericardial rind demonstrated organizing fibrin with cholesterol cleft granuloma (*Figure 4*). Fungal and bacterial culture of the pericardial fluid including Acid-fast bacilli of the pericardial fluid did not have any growth. QuantiFERON-TB Gold test was negative for mycobacterium tuberculosis infection. Although pericardial fluid analysis for cholesterol and triglyceride content was sent, it was not performed by laboratory. He initiated colchicine therapy 0.6 mg twice daily and completed one month of therapy. The patient had an unremarkable postoperative course and remained symptomatically improved at >1 year of follow-up without recurrence of the pericardial effusion.

## Discussion

Cholesterol-rich pericardial effusions are rarely reported in the literature; when they are, they typically present as large, chronic effusions.^3^ Our case demonstrates an atypical presentation. CMR demonstrated no pericardial inflammation, and revealed a loculated and likely exudative pericardial effusion rather than a circumferential effusion. The loculated effusion caused constrictive physiology. Despite drainage of the effusion with pericardial window, there was no improvement in haemodynamic ultimately necessitating partial local pericardiectomy and cruciate incision of the anterior aspect of the rind of thickened pericardium.

Various cases in literature associated RA with the development of cholesterol pericarditis.^5^ Interestingly, RA susceptibility genes such as TRAF1/C5, STAT4, and HLA-DRB1 shared epitope have been associated with altered lipid levels in patients with RA, potentially contributing to more rapid development of coronary artery disease.^6,7^ It is unclear whether similar abnormal lipid metabolism in RA that contributes to accelerated atherosclerosis may also contribute to development of cholesterol pericardial effusion—although this is not among suggested hypotheses.^1^ Our patient demonstrated a consistent decrease in HDL; 33 mg/dL prior to his operation compared with 44 mg/dL and 54 mg/dL 2 months before. This paradoxical decrease, along with his generally lower lipid panel values prior to operation are suggestive of the ‘lipid paradox,’ described in literature, where HDL values decrease, in different disease states of RA and increasing systemic inflammation.^8^ This aligns with our understanding that his cholesterol effusion, developed in a subacute fashion.

Our patient had been had a small pericardial fluid collection felt to be a pericardial cyst one month prior without features of constriction. We propose that the combination of RA-driven dyslipidemia and anticoagulation-induced microbleeding (anticoagulation for prior diagnosis of pulmonary embolism) promoted the development of a cholesterol-rich loculated pericardial effusion. This hypothesis is supported by pathology, which demonstrated fibrin-rich, cholesterol-laden granuloma. Although the laboratory did not perform analysis of the pericardial fluid for triglyceride and cholesterol content as requested to assess for the possibility of chylopericardium, the present of cholesterol cleft granuloma is diagnostic for cholesterol pericarditis and not typically seen in chylopericardium. While there is literature that indicates the risk of haemorrhagic transformation of pericardial effusion in the setting of anticoagulation, in our case, we propose that microbleeding *and* the RA driven milieu for lipid pathology allowed the condition for a loculated effusive-constrictive process to manifest. This two-hit hypothesis is supported by the lack of pericardial inflammation on CMR suggesting that inflammatory mechanism from just RA was unlikely in isolation.

This case underscores the need to consider cholesterol pericardial effusion with constrictive features in RA patients—even in the absence of overt pericardial inflammation on imaging. In our case, the use of MRI was critical in differentiating between a simple transudative pericardial effusion and the cholesterol-laden fluid collection we discovered. While echocardiograms and CT showcase the anatomical delineation of the pericardial fluid collection and the haemodynamic effects well, MRI allowed further tissue characterization with the use of T1-weighted, T2-weighted, and LGE sequences. Given the complimentary information from multimodality imaging, we were able to anticipate that unlike for a pericardial cyst or simple transudative pericardial effusion, drainage of the fluid collection alone was unlikely to resolve the constrictive physiology. Despite the inherent risks of pericardiectomy, including a 6–12% operative mortality reported in literature, partial local pericardiectomy and cruciate incision of the anterior aspect of the rind was essential in relieving the constrictive process.^9^

## Lead author biography


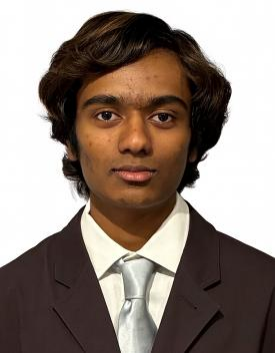


Aadavan Elangovan is a senior undergraduate student pursuing a Microbiology and Cell Science degree at the University of Florida (UF), on a pre-medicine track. Additionally, Aadavan currently works on the cardiac floor at UF Shands Hospital as a telemetry technician and patient care assistant. Outside of clinical and academic pursuits, he has pursued service and philanthropy, raising over $15,000 dollars towards the American Heart Association through hospital-based initiatives-a record holder for individual fundraising at the UF Heart and Vascular Institute. He plans to apply to medical school with aspirations of becoming a cardiologist. His current specific interests are in advanced cardiac imaging and cardiovascular research.

## Supplementary Material

ytag261_Supplementary_Data

## Data Availability

The data underlying this article are available in the article and in its online supplementary material.
