# Supplementary material for: A cheesy exudate: a case report on management of pericardial disease through multimodal cardiac imaging
Source: Eur Heart J Case Rep. 2026 Apr 16;10(4):ytag261. doi: 10.1093/ehjcr/ytag261 (PMC13128196; doi:10.1093/ehjcr/ytag261)
Supplement: ytag261_Supplementary_Data [file ytag261_supplementary_data.zip › Video captions.docx]

**Video 1:** Parasternal short axis echo demonstrating compression of the right sided chambers by an echolucent fluid containing mass most prominent adjacent to the tricuspid valve annulus (yellow arrows).

**Video 2:** Apical 4-chamber view echo demonstrating respiratory variation in the ventricular septal motion suggestive of ventricular interdependence and constrictive physiology is also seen. Compression of the mitral valve inflow adjacent to the lateral mitral valve annulus is also visualized (red arrow).

**Video 3:** Intraoperative TEE prior to drainage of the pericardial fluid collection demonstrating a heterogenous pericardial fluid collection (A; yellow arrow) adjacent to the right sided heart chambers with compressive mass effect on the chambers.

**Video 4:** Intraoperative TEE after pericardial fluid collection drainage, compressive mass effect on the right-sided chambers by the pericardial mass (red arrow) was still visualized.

**Video 5:** Intraoperative TEE images demonstrated improved appearance of the compression of the right sided chambers (red arrow) following pericardiectomy.
